# Influence of Plasma Processing on Recovery and Analysis of Circulating Nucleic Acids 

**DOI:** 10.1371/journal.pone.0077963

**Published:** 2013-10-18

**Authors:** Karen Page, David S. Guttery, Nathalie Zahra, Lindsay Primrose, Shona R. Elshaw, J. Howard Pringle, Kevin Blighe, Stephanie D. Marchese, Allison Hills, Laura Woodley, Justin Stebbing, R. Charles Coombes, Jacqueline A. Shaw

**Affiliations:** 1 Department of Cancer Studies and Molecular Medicine, University of Leicester, Leicester Royal Infirmary, Leicester, United Kingdom; 2 Division of Cancer, ICTEM, Hammersmith Campus, London, United Kingdom; The University of Tokyo, Japan

## Abstract

Circulating nucleic acids (CNAs) are under investigation as a liquid biopsy in cancer. However there is wide variation in blood processing and methods for isolation of circulating free DNA (cfDNA) and microRNAs (miRNAs). Here we compare the extraction efficiency and reproducibility of 4 commercially available kits for cfDNA and 3 for miRNA using spike-in of reference templates. We also compare the effects of increasing time between venepuncture and centrifugation and differential centrifugation force on recovery of CNAs. cfDNA was quantified by TaqMan qPCR and targeted deep sequencing. miRNA profiles were assessed with TaqMan low-density arrays and assays. The QIAamp^®^ DNA Blood Mini and Circulating nucleic acid kits gave the highest recovery of cfDNA and efficient recovery (>90%) of a 564bp spike-in. Moreover, targeted sequencing revealed overlapping cfDNA profiles and variant depth, including detection of *HER2* gene amplification, using the Ion AmpliSeq™Cancer Hotspot Panel v2. Highest yields of miRNA and the synthetic *Arabidopsis thaliana miR-159a* spike-in were obtained using the miRNeasy Serum/Plasma kit, with saturation above 200 µl of plasma. miRNA profiles showed significant variation with increasing time before centrifugation (p<0.001) and increasing centrifugation force, with depletion of platelet associated miRNAs, whereas cfDNA was unaffected. However, sample replicates showed excellent reproducibility on TaqMan low density arrays (ρ = 0.96, p<0.0001). We also successfully generated miRNA profiles for plasma samples stored > 12 years, highlighting the potential for analysis of stored sample biobanks. In the era of the liquid biopsy, standardisation of methods is required to minimise variation, particularly for miRNA.

## Introduction

There is wide interest in the utility of circulating nucleic acids (CNAs) as biomarkers of cancer. Thus far, the majority of research has focussed on circulating cell-free DNA (cfDNA) due to its stability and ease of isolation. Increasing quantities are found in cancers compared to healthy controls, with high levels sometimes in excess of 100 ng/ml detected in advanced disease [1]. 

So far there has been no standardisation of methods for cfDNA isolation, making comparison between different studies and markers difficult. In addition, recent studies have correlated a large proportion of tumour-specific aberrations with low-molecular weight fractions of cfDNA [2,3]. This highlights the need for optimum protocols for extraction of cfDNA to ensure minimal contamination by high-molecular weight DNA fragments from stromal, inflammatory, and mononuclear cells [4], that might influence downstream analyses such as deep sequencing.

Circulating microRNAs (miRNAs) are also increasingly under investigation [5]. These miRNAs are small regulatory molecules, which function as negative regulators of target genes by directing specific mRNA cleavage or inhibition of translation. There are 2,042 mature human miRNAs described in miRBase V19 (http://www.mirbase.org/) and miRNA sequences are being integrated with deep sequencing data [6]. Recent studies have identified tumour–associated miRNAs, elevated in plasma of cancer patients [5,7] and have shown that these are stable, protected from endogenous RNAse activity [8] in micro-vesicles [9], and are also well preserved in formalin-fixed paraffin embedded (FFPE) tissues. 

A number of methods have been used to isolate cfDNA from plasma, ranging from simple boiling [10], SDS/Proteinase K digestion and phenol/chloroform extraction, to commercially available small-scale column-based extraction kits and magnetic bead technology. In our previous studies of cfDNA in patients with breast cancer, we have routinely used the QIAamp^®^ DNA Blood Mini Kit for isolation of cfDNA [11-14]. Similarly, there are an increasing number of commercial kits marketed for isolation of miRNA. 

The aim of this study was to investigate the effects of pre-analytical processing on isolation of CNAs from plasma, and also to compare the extraction efficiency, yield and reproducibility obtained using commercially available kits using spike-in of reference templates. We compared 4 kits for cfDNA isolation (QIAamp^®^ DNA Blood Mini, QIAamp^®^ Circulating Nucleic Acid Kit, NucleoSpin^®^ Plasma XS kit and FitAmp^®^ Plasma/Serum DNA Isolation kit), and 3 kits for miRNA isolation (*miRvana* microRNA Isolation kit, miRNeasy Serum/Plasma kit and QIAamp^®^ Circulating Nucleic Acids kit). Yields of cfDNA and miRNA and recovery of spike-in references varied widely between the different kits. miRNA yield was significantly affected by both centrifugation of plasma at 10000g, which depletes platelets and leaving blood at room temperature for > 2 hours, whereas cfDNA was unaffected. Moreover, sequencing of cfDNA samples with Ion AmpliSeq™Cancer Hotspot Panel v2 recovered using the “top 2” kits showed no significant difference in the coverage of the 207 amplicons in the panel. Finally, we suggest standard approaches for optimum blood processing and isolation of cfDNA and circulating miRNAs.

## Materials and Methods

### Ethics statement

The study protocol was approved by the Riverside Research Ethics Committee (Imperial College Healthcare NHS Trust; REC reference number: 07/Q0401/20) and the Leicestershire, Northamptonshire & Rutland Research Ethics Committees (REC reference number: 05/Q2502/28; 11/EM0023). Blood sample collection was conducted in accordance with the Declaration of Helsinki. All patients gave written informed consent prior to participation. 

### Blood processing

20 ml venous blood samples were drawn in the morning into EDTA-containing tubes (BD Biosciences) and centrifuged at 1000g (Jouan CR422; Jouan) for 10 min at 4 °C. Plasma was carefully removed, leaving 3-5 mm above the buffy coat, added to a 15 ml Falcon tube (BD Biosciences) and re-centrifuged at 1000g for 10 min at 4 °C. Plasma was carefully removed and stored at -80 °C in 1 ml aliquots in 1.5 ml tubes (Starlab) for cfDNA, and in 1.5 ml RNase-free tubes (Starlab) for miRNA. We also varied the speed of the second centrifugation step: 1000g, 2000g (Jouan CR422; Jouan) or 10000g (Microcentaur; MSE) each for 10 min at 4 °C, as well as increasing the time between venepuncture and the first centrifugation step (at 2 hr and at 6 hr). On thawing a third and final spin was performed at 1000g for 5 min at room temperature and plasma carefully removed into a separate tube away from any residual debris before extraction of CNAs.

### Extraction of circulating nucleic acids, spike-in templates and QC checks

We purchased 3 plasma samples from healthy females (age range 35 - 39 years) (Seralab) and pooled an equal volume to generate a reference healthy plasma sample. For cfDNA we compared isolation from 1 ml starting plasma volume, with each sample/kit performed in triplicate, using the QIAamp^®^ DNA Blood Mini Kit, QIAamp^®^ Circulating Nucleic Acid Kit, NucleoSpin^®^ Plasma XS Kit (Macherey-Nagel) and FitAmp^®^ Plasma/Serum DNA Isolation kit (Epigentek). Each extraction was performed exactly according to manufacturers’ instructions and as described previously [12,13]. With each sample/kit the elution volume was 1/10th of the starting plasma volume. As additional validation we spiked λ/*Hind*III DNA (Ambion) into 1 ml of the pooled healthy plasma at 4 fixed concentrations (50 ng, 5 ng, 0.5 ng and 0.05 ng) and assessed recovery of two fragments by real-time qPCR. 

Circulating miRNAs were extracted from 1 ml, 500 µl or 200 µl of plasma. As miRNA was not recovered from the pooled healthy plasma (Seralab) we isolated miRNA from pooled plasmas from 3 patients with either primary or metastatic breast cancer. Each extraction was performed in triplicate, using the miRNeasy Serum/Plasma Kit (Qiagen), *miRvana* microRNA Isolation kit (Ambion) or QIAamp^®^ Circulating Nucleic Acid Kit (Qiagen), according to the manufacturers’ instructions. 30 pg or 30 ng of the synthetic *Arabidopsis thaliana miR-159a* (*ath-miR-159a*; 5’-UUUGGAUUGAAGGGAGCUCUA-3’) (Integrated DNA Technologies) was spiked-in to each sample after the initial lysis step to test extraction efficiency. All samples were eluted in 50 µl of RNase-free water.

Initial QC checks of eluted cfDNA and RNA (1 µl each) were performed using the Qubit® 2.0 dsDNA high sensitivity assay (Life Technologies) and Agilent Bioanalyser 2100 analysis with RNA 6000 Pico reagents and chips (Agilent), respectively, according to manufacturers instructions.. 

### Reverse transcription and analysis of miRNA profiles

MicroRNAs were reverse transcribed from 3 µl of sample using the TaqMan Megaplex RT system (Applied Biosystems), and pre-amplified from 2.5 µl of cDNA using the TaqMan Megaplex Pre-Amp system (Applied Biosystems) according to manufacturer’s instructions on a Veriti thermal cycler (Applied Biosystems). Pre-amplified cDNA was diluted 1:25 prior to use and compared with unamplified cDNA, diluted 1:5 in 1xTE buffer. A RT-negative control was included in each experiment to assess genomic DNA contamination, all of which showed no amplification upon analysis by qPCR for *hsa-miR-21*. For validation of the TLDA cards, pre-amplified cDNA was diluted 1:100. 

For miRNA PCR arrays, TLDA v2.0 cards were used according to manufacturer’s instructions on an Applied Biosystems 7900 thermal cycler as follows: 50 °C for 2 min, 94.5 °C for 10 min, followed by 40 cycles of 97 °C for 30 sec and 59.7 °C for 1 min. The cycle threshold (C_T_) values were determined using a fixed threshold of 0.1 for all qPCR experiments. Exiqon miRCURY LNA Serum/Plasma focus panels were analysed on a Roche Lightcycler 480 thermal cycler as follows: 95 °C for 10 min, followed by 45 cycles of 95 °C for 10 sec and 60 °C for 1 min with a ramp rate of 1.6 °C/sec.

MiRNA samples were analysed in triplicate using 3 µl of diluted template and TaqMan microRNA assays (*hsa-miR-191* (assay code 000490), *hsa-miR-21* (000397), *ath-miR-159a* (000338), *hsa-miR-15b* (000390), *hsa-miR-16* (000391), *hsa-miR-24* (000402), *hsa-miR-155* (002623) or *hsa-miR-484* (001821) (Applied Biosystems)) according to manufacturer’s instructions. Cycling was performed using a 10 µl reaction on a Step 1 Plus thermal cycler: for cfDNA: 95 °C for 20 sec, followed by 50 cycles of 95 °C for 1 sec and 60 °C for 20 sec; for miRNA assays: 95 °C for 20 sec, followed by 40 cycles of 95 °C for 1 sec and 60 °C for 20 sec. Results were analysed with Step 1 v2.2 software and Microsoft® Excel.

### Quantitation of cfDNA and Targeted Deep Sequencing

Each sample (3.6 µl of eluted cfDNA) was quantified in triplicate relative to a standard curve, generated from a serially-diluted template (Human Genomic DNA; Roche) of known concentration, and converted to ng/mL using the following equation: (Total quantity of cfDNA/3.6) x 1000, and an in-house locus-specific TaqMan assay targeting *GAPDH* [11], and two inventoried assays for *ACTBL2* (Hs001101944_s1, targeted amplicon 67 bp) and *HPRT1* (Hs03929096_g1, targeted amplicon 64 bp) (Applied Biosystems). For the λ/*Hind*III DNA spike-in experiments, two fragments (23kb and 564 bp) were quantified using custom primers (Sigma) and FAM-MGB probes (Applied Biosystems). The spike-in was quantified in triplicate relative to a standard curve generated from serially-diluted λ/*Hind*III DNA. Sequences were: 23 kb fragment: forward, 5’-CGCACAGGAACTGAAGAATG-3’; reverse, 5’-CCGTCGAGAATACTGGCAAT-3’; FAM-MGB probe, 5’-TGTACTTTCGTGCTGTCGCGGATCG-3’ (targeted amplicon 108 bp); 564 bp fragment: forward, 5’-TGGGATCATTGGGTACTGTGG-3’; reverse, 5’-CAGACTTGGGGGTGATGAGTTT-3’; FAM-MGB probe, 5’-CCGCTATCCCTGATCAG-3’ (targeted amplicon 97 bp). 

For DNA sequencing, cfDNA was isolated from 1 ml plasma of four patients with metastatic breast cancer, using two kits (QIAamp^®^ DNA Blood Mini Kit and the QIAamp^®^ Circulating Nucleic Acid Kit) to generate 8 templates. 10 ng of each template was sequenced in pairs on two 316 chips on the Ion PGM™ Sequencer using the Ion AmpliSeq™Cancer Hotspot Panel v2 according to manufacturer’s instructions. DNA sequencing data was accessed through the Torrent Suite v.3.4.2. Reads were aligned against the human genome (hg19) using Alignment v 3.4.48996 and variants called using the coverageAnalysis v 3.4.51836 and variantCaller v 3.4.51874, respectively. All raw BAM files have been deposited at the European Nucleotide Archive (ENA - https://www.ebi.ac.uk/ena) under accession number PRJEB4510.

### Statistical analysis

All statistical analysis was performed using GraphPad Prism 6 for Windows. An unpaired Mann-Whitney test was used for comparison of RNA extractions and a two-tailed Spearman’s correlation analysis was used for correlation analysis of the TLDA and miRCURY cards. Where indicated, miRNA ΔC_T_ values were normalised to the average C_T_ values obtained on the miRCURY PCR array and TLDA card A before cycle 35 [15]. A matched-paired Wilcoxon test was used to compare the variants detected in the cfDNA sequenced on the Ion PGM™ Sequencer using the Ion AmpliSeq™Cancer Hotspot Panel v2. 

## Results

### Recovery of cfDNA and λ/*Hind*III spike-in

**Figure 1:**
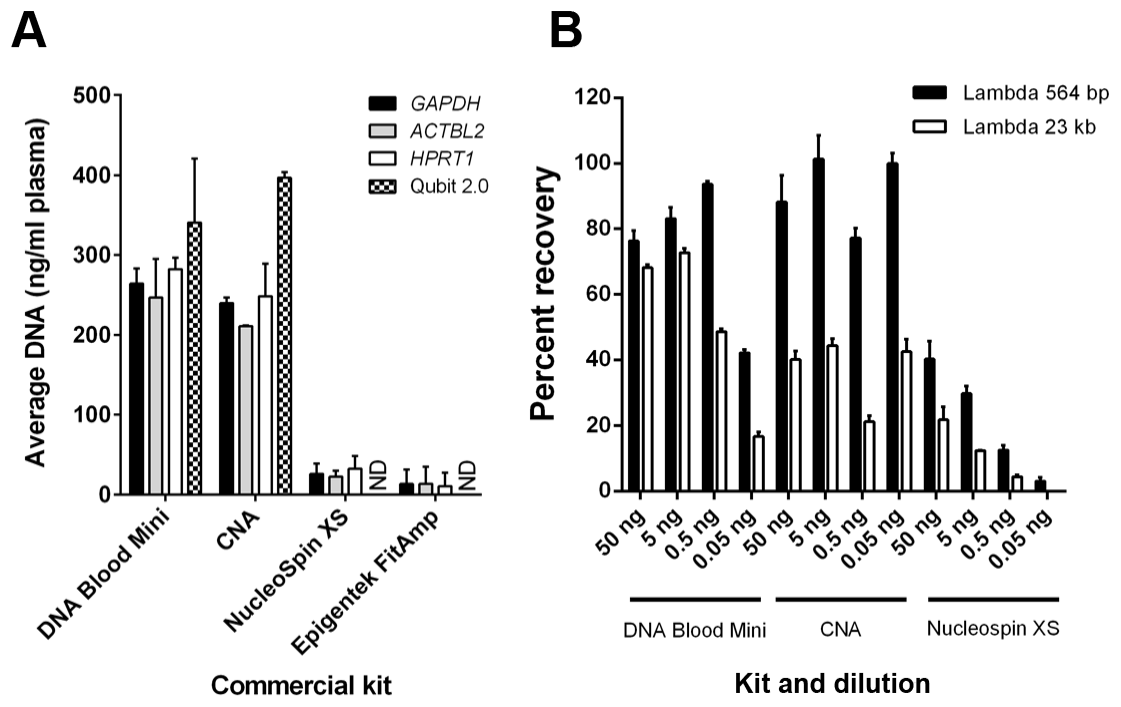
Comparison of cfDNA recovery using different commercially available kits. A. Average cfDNA yield per 1 ml of plasma with 4 extraction kits, quantified using qPCR for *GAPDH*, *ACTBL2* and *HPRT* and Qubit® 2.0 High Sensitivity dsDNA Assay. Error bar = ±SEM, n = 9 from 3 independent experiments each performed in triplicate. ND = Not detectable. B. Percent recovery of serial dilutions of λ/*Hind*III DNA, quantified using assays targeting the 23 kb and 564 bp fragments. Error bar = ±SEM, n = 9 from 3 independent experiments performed in triplicate. CNA = Circulating Nucleic Acid kit.

We compared 4 commercial kits for isolation of cfDNA, using 1 ml of pooled healthy plasma. Each extraction was performed in triplicate and each cfDNA sample was quantified in triplicate by qPCR targeting *GAPDH* [13], *ACTBL2* and *HPRT1*. The 3 assays showed equivalent results, with the QIAamp^®^ DNA Blood Mini and QIAamp^®^ CNA kits giving the highest quantities of cfDNA/ml of plasma (mean 264.28 ng/ml and 239.85 ng/ml, respectively), 10 fold higher than the other two kits (Figure 1A). As validation, we also performed Qubit® 2.0 analysis, which confirmed this trend but with a slightly higher recovery of cfDNA (Figure 1A).

To determine the efficiency of cfDNA recovery, we assessed recovery of spiked-in λ/*Hind*III DNA using the 3 kits that gave the highest yields of cfDNA (QIAamp^®^ DNA Blood Mini Kit, QIAamp^®^ CNA Kit and NucleoSpin^®^ Plasma XS Kit). The QIAamp^®^ DNA Blood Mini kit and the QIAamp^®^ CNA Kit showed similar but variable recovery of the 23kb fragment (range 20-70%). The QIAamp^®^ CNA Kit gave the highest yield of the smaller (564 bp) fragment, recovering between 90 - 100% at each dilution tested; whereas the NucleoSpin^®^ Plasma XS Kit showed a lower recovery of both amplicons (Figure 1B). 

### cfDNA extraction methods generate equivalent targeted deep sequencing data

**Figure 2:**
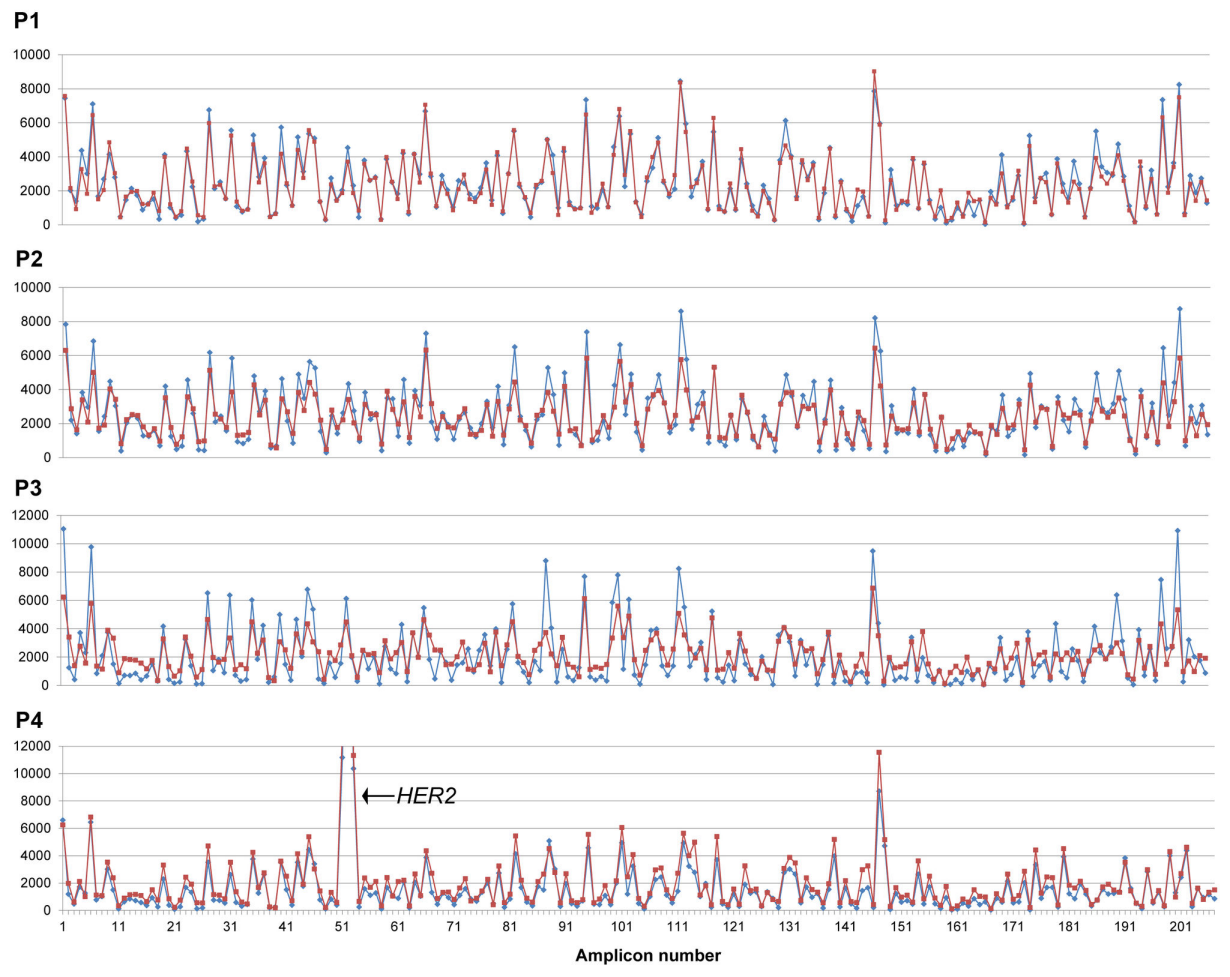
Comparison of number of reads between two different extraction kits using the Ion AmpliSeq™Cancer Hotspot Panel v2. Analysis of 4 plasma samples extracted using both the QIAamp^®^ DNA Blood Mini kit and the QIAamp^®^ CNA Kit. In each trace, the number of reads is shown on the Y-axis. Red lines indicate the QIAamp^®^ CNA Kit and the blue lines represent the QIAamp^®^ DNA Blood Mini kit. For in depth analysis see Tables S1 and S2.

To determine whether the two kits giving the best recovery of cfDNA (QIAamp^®^ DNA Blood Mini kit and the QIAamp^®^ CNA Kit) showed any variance in cfDNA profiles, paired cfDNA from 4 metastatic patients were sequenced using the Ion AmpliSeq™Cancer Hotspot Panel v2 on the Ion PGM™ Sequencer. A total of 412M bases were generated across the eight cfDNA samples, with an average of 52 ± 5 M bases per sample (Table S1). This was equivalent to 503 ± 46K reads, > 96% of which mapped to the target region providing an average coverage of 2000x. High similarity was observed in the coverage profile for each cfDNA pair across the 207 amplicons (average size 154 bp, range 111bp -187 bp) (Figure 2). Of note, one sample (P4) showed a peak in the number of reads (>30,000 reads) at *ERBB2* suggesting gene amplification. The total number of reads at each amplicon showed high correlation, (ρ > 0.95, *p*<0.001) for 3 of the 4 cfDNA pairs (Table S1). In the one sample (P3) with lower but significant correlation (ρ = 0.87) the QIAamp^®^ CNA method gave a more uniform coverage that the QIAamp^®^ DNA Blood Mini method. 

A total of 167 germline and somatic variants were called with an average of 21 variants per sample (Table S2). 136 (81%) of the total variants were matched in paired samples in terms of variant position and frequency with no significant difference in percentage variant call (p>0.05, *p*<0.001). Thirty-one (19%) variants were unique, but the majority of these were in P3 and detected at <10% depth. 

### Recovery of circulating miRNA

**Figure 3:**
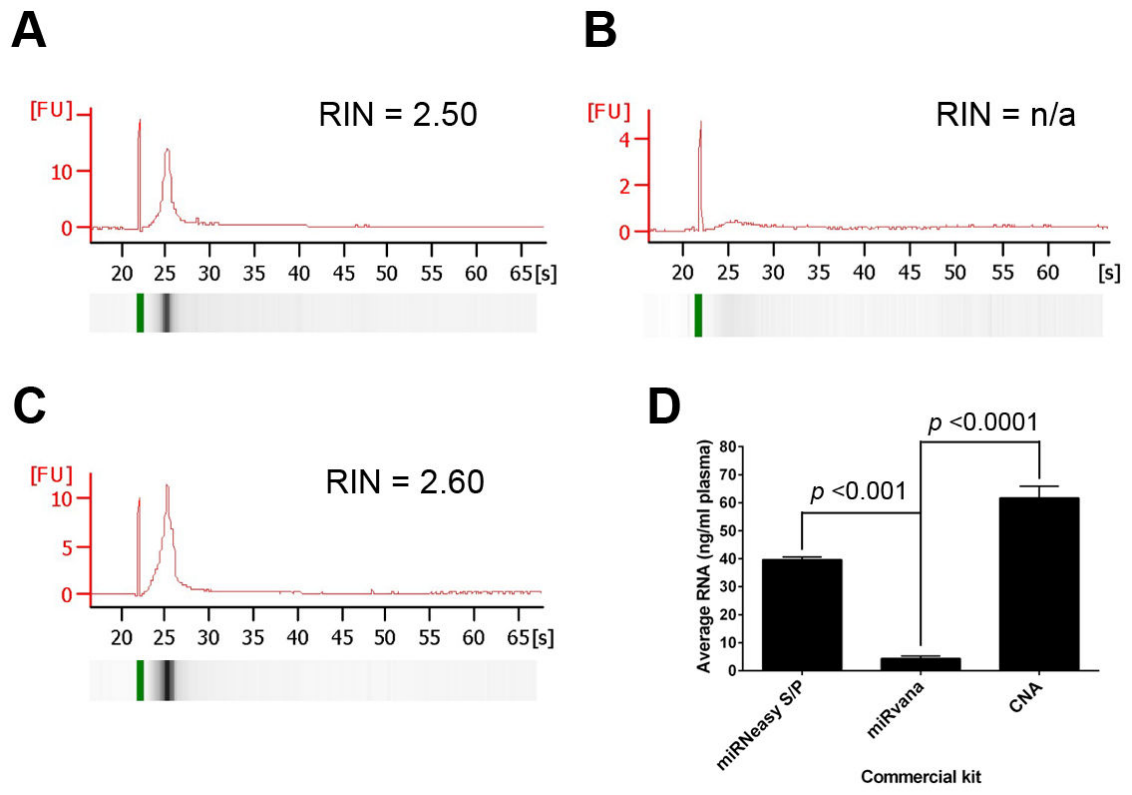
Bioanalyzer analysis of circulating miRNA iolated with different kits. A-C. Representative Bioanalyser traces of cell-free RNA obtained from 1 ml of plasma using 3 commercial kits. The kits used and RNA integrity (RIN) values are highlighted above the trace and representative gels are shown below each trace. A = extractions using the miRNeasy Serum/Plasma kit, B = *mirVana* microRNA Isolation kit and C = Circulating Nucleic Acid kit. Quantities obtained are shown in D. Error bar = ±SEM, n = 3 from 3 independent experiments.

**Figure 4:**
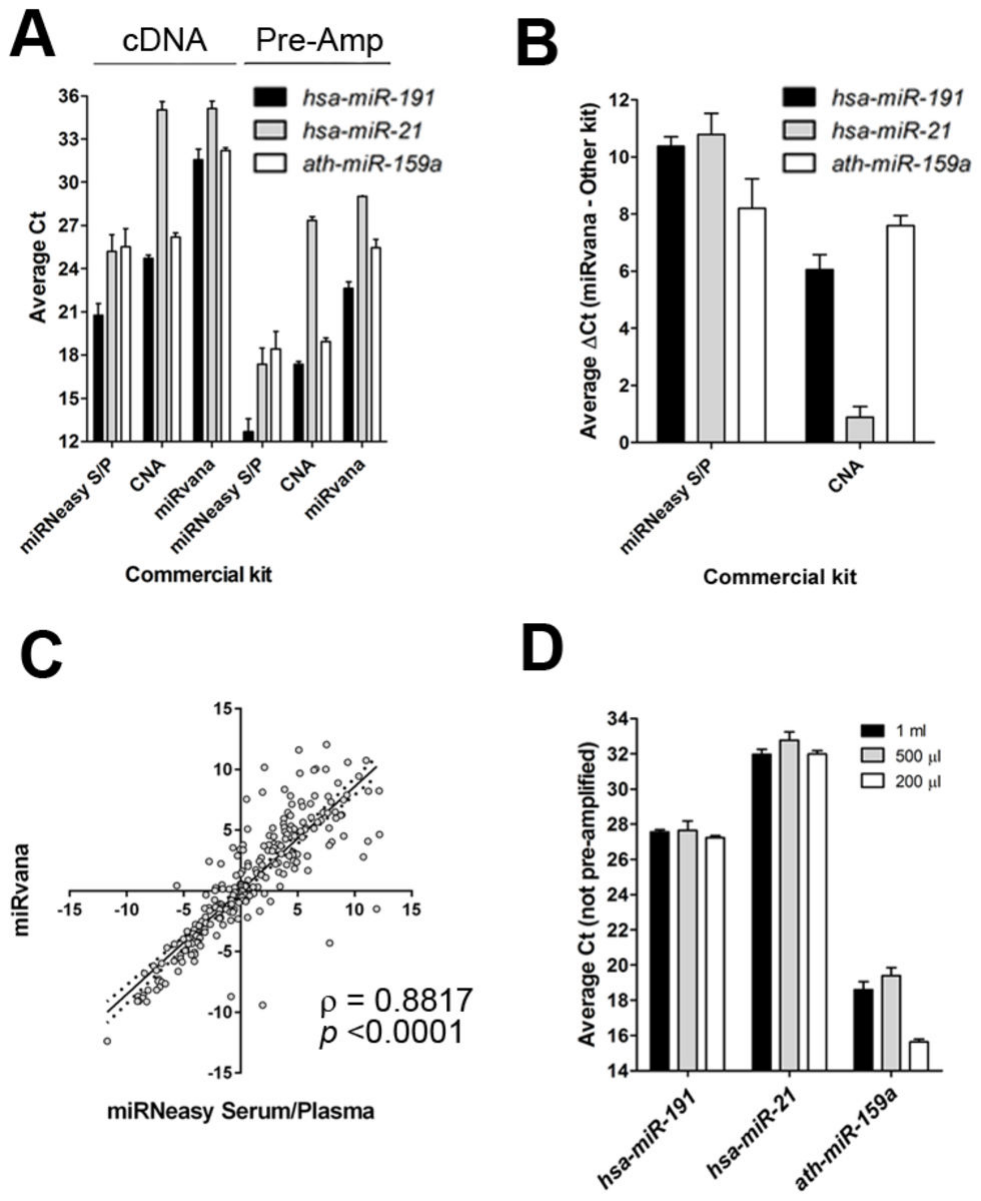
qRT-PCR analysis of miRNA isolated with different kits. A. Average C_T_ values obtained for *hsa-miR-191, hsa-miR-21* and *ath-miR-159a* on cDNA and pre-amplified cDNA (Pre-Amp) from 1 ml of plasma. Error bar = ±SEM, n = 9 from 3 independent experiments performed in triplicate. S/P = Serum/Plasma kit, CNA = Circulating Nucleic Acid kit. B. Average ΔC_T_ values comparing the miRNeasy Serum/Plasma and CNA kit to the *miRvana* Isolation kit. ΔC_T_ values calculated using the equation: Average miRNA C_T_ using the *miRvana* kit - Average miRNA C_T_ using another kit (i.e miRNeasy Serum/Plasma or CNA kit). Error bar = ±SEM, n = 9 from 3 independent experiments performed in triplicate. S/P = Serum/Plasma kit, CNA = Circulating Nucleic Acid kit. C. Two-tailed Spearman’s correlation analysis of ΔC_T_ values obtained from TLDA A cards and samples extracted from 1 ml of plasma using the *miRvana* Isolation kit or 200 µl of plasma using the miRNeasy Serum/Plasma kit. Dotted lines represent the 95% confidence interval. Rho (ρ) and *p*-values are indicated on the graph. D. Average C_T_ values obtained analysing *hsa-miR-191, hsa-miR-21* and *ath-miR-159a* in cDNA produced from 1 ml, 500 µl or 200 µl of plasma using the miRNeasy Serum/Plasma kit. Error bar = ±SEM, n = 9 from 3 independent experiments performed in triplicate.

We compared 3 commercial kits for extraction of circulating miRNA. Unfortunately, none were successful at recovering miRNA from 1 ml of the pooled control plasma, as two miRNAs (*hsa-miR-21* or *hsa-miR-191*) were undetected by qPCR. Therefore, we trialled the 3 kits using pooled plasma from 3 primary breast cancer patients recruited to our studies. There were marked differences in recovery of miRNA between the three kits. Bioanalyser analysis showed minor differences in miRNA yields for the QIAamp^®^ CNA or miRNeasy Plasma/Serum kit; which were significantly higher than the *miRvana* microRNA Isolation kit (*p* <0.001) (Figure 3). Considering individual miRNAs, qRT-PCR revealed lower C_T_ values (4 - 10 cycles earlier) for both *hsa-miR-21* and *hsa-miR-191* with the miRNeasy Serum/Plasma kit compared to the QIAamp^®^ CNA and *miRvana* kits when analysing both cDNA and pre-amplified cDNA (Figure 3A, B). The C_T_ values for the synthetic *ath-miR-159a* spike-in were also consistently lower using the miRNeasy Serum/Plasma kit compared to the *miRvana* kit, but not to the QIAamp^®^ CNA kit (Figure 4A, B). 

Of note, although the *miRvana* microRNA Isolation kit gave the lowest yield of miRNAs, even from 1 ml of pooled plasma, the profile of miRNAs detected by TLDA cards showed significant correlation with the profile generated following extraction of miRNA from 200 µl of plasma using the miRNeasy Serum/Plasma kit (*p* <0.0001) (Figure 4C). Based on these results, further optimisation of miRNA isolation was only performed using the miRNeasy Serum/Plasma kit. 

### The yield of miRNA is not dependent on plasma volume

Next, we compared miRNA yields from 200 µl, 500 µl and 1 ml starting plasma volume. There was no significant increase in miRNA yield with increasing plasma volume by either Bioanalyser analysis (Figure S1) or qRT-PCR (*hsa-miR-21* and *hsa-miR-191*) (Figure 4D), suggesting the quantity of miRNA recovered was not volume dependent. As part of this experiment, we also spiked-in *ath-miR-159a* at a concentration of 30 ng (1000-fold higher than in the previous kit comparisons) to test whether this resulted in saturation of either the column or the qPCR. Although the 1000-fold increase in concentration of the spike-in was reflected by an appropriate C_T_ value with 200 µl plasma volume, there was an increase in C_T_ values (and therefore decrease in detected concentration of the spike-in) with increasing plasma volume, suggesting less efficient recovery. 

### Pre-analytical processing alters the yield of miRNA but not cfDNA

**Figure 5:**
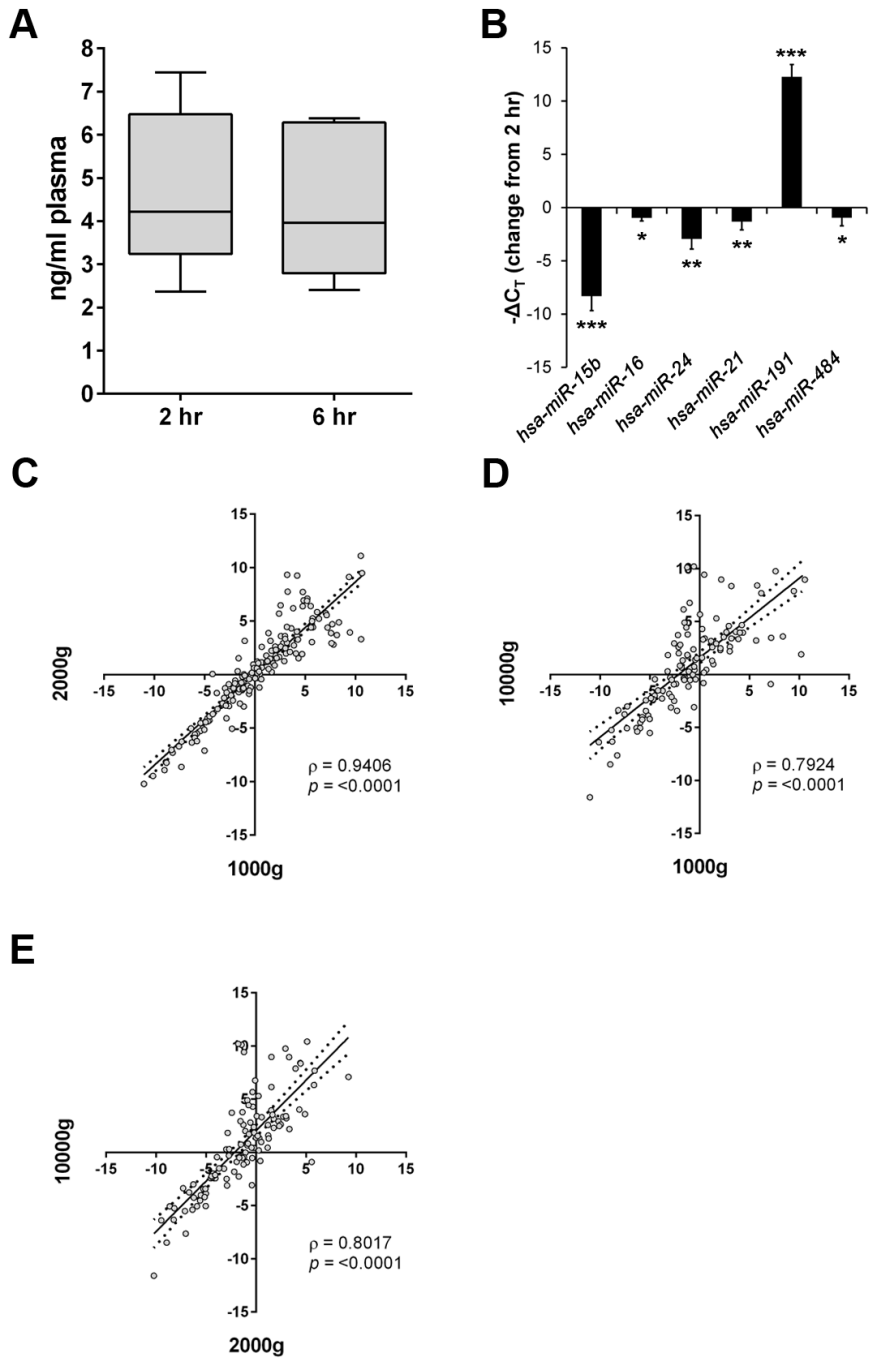
Recovery of cfDNA and miRNA from plasma: effects of increasing centrifugation speed. A. Quantitation of cfDNA using qPCR for *GAPDH*. Blood was processed within 2 hr or 6 hr post-venepuncture and cfDNA isolated from 1 ml of plasma. Whiskers of the box-plot show minimum and maximum values. B. Comparison of miRNA profiles from plasma processed 6 hr post-venepuncture compared to samples processed at 2 hr. Results presented as -ΔC_T_ (change from 2 hours. Error bar = ±Std Dev, n = 5. *p<0.05; **p<0.01; ***p<0.001. C. Two-tailed Spearman’s correlation analysis of ΔC_T_ values obtained from TLDA A cards for duplicate miRNA samples following centrifugation at 1000g and 2000g. Dotted lines represent the 95% confidence interval. D. Two-tailed Spearman’s correlation analysis of ΔC_T_ values obtained from TLDA A cards for duplicate miRNA samples following centrifugation at 1000g and 10000g. Dotted lines represent the 95% confidence interval. E. Two-tailed Spearman’s correlation analysis of ΔC_T_ values obtained from TLDA A cards for duplicate miRNA samples following centrifugation at 2000g and 10000g. Dotted lines represent the 95% confidence interval. For C - E, Rho (ρ) and *p*-values are indicated on each graph.

We obtained 15 ml blood samples from 5 healthy female volunteers and processed 7.5 ml for plasma at 2 hours post-venepuncture, while the remaining 7.5 ml was left for 6 hours at room temperature before processing. Plasma was separated using the 2 step 1000g, 1000g protocol (see methods). Both cfDNA and miRNA were recovered in triplicate from 1 ml and 200 µl of plasma using the QIAamp^®^ DNA Blood Mini kit and miRNeasy Serum/Plasma kit, respectively and analysed by qPCR. Yields of cfDNA were not altered by blood processing time (Figure 5A); however, 6 candidate miRNAs analysed were significantly different, in particular, *hsa-miR-15b* and *hsa-miR-191* (*p*<0.001) (Figure 5B). 

We also compared recovery of cfDNA and miRNA from 1 ml and 200 µl of pooled plasma, respectively following centrifugation at 1000g then either 1000g, 2000g or 10000g. Yields of cfDNA were equivalent, as assessed by *GAPDH* quantitation (data not shown); however, miRNA yield was notably lower after centrifugation at 10000g. Following profiling with TLDA card A, there were a total of 195 miRNAs detected after 1000g and 187 miRNAs at 2000g, but only 138 miRNAs detected at 10000g (Table S3). However, comparison of ΔC_T_ values showed significant correlation between these (Figure 5C-E), particularly between samples centrifuged at 1000g and 2000g. As expected, platelet-associated miRNAs including *hsa-miR-24, hsa-miR-191, hsa-miR-197* and *hsa-miR-223* [16] were notably reduced after centrifugation at 10000g (Table S3).

### MiRNA profiles show good reproducibility between replicate samples

To test reproducibility of the miRNA PCR arrays, we analysed pooled samples from patients with primary breast cancer in duplicate on both TLDA cards and the Exiqon miRCURY LNA microRNA PCR array. Both platforms showed high reproducibility between replicate samples (Figure S2A, B). There was also a lower but significant correlation between miRNA profiles on the two platforms (ρ = 0.6, *p*<0.0001 - Figure S2C). Furthermore, as melt-curve analysis showed that a number of positive probes with high C_T_ values (>35) on the miRCURY LNA platform had multiple melting temperatures (Figure S2C); further experiments were performed using the TLDA cards only. 

### Recovery of miRNA from stored plasma

Our final aim was to determine whether miRNA was recoverable from stored plasma, as we have shown previously that cfDNA can be isolated and analysed from plasma samples stored for > 12 years [14]. To achieve this we compared 20 plasma samples from patients with metastatic breast cancer as two pools: 10 extracted from freshly frozen plasma and 10 from plasma stored at -80 °C for >12 years. A total of 177 miRNAs were detected in the stored pool, compared to 202 miRNAs in the fresh pool (TLDA Pool A v2.0 Cards, Table S4), suggesting some loss of miRNAs on storage. We assessed whether C_T_ values were lower in fresh compared to stored samples and found no significant difference (*p* = 0.77). However, there was a significant correlation of both C_T_ and ΔC_T_ values between miRNAs detected in the two pools (ρ = 0.84, *p*<0.0001, and ρ = 0.82, *p*<0.0001, respectively – Figure S2D, E), suggesting similar yield of miRNA and confirming successful recovery of miRNA from long term stored plasma. 

## Discussion

There is currently no consensus protocol for pre-analytical processing of blood for isolation of CNAs. Ideally, blood samples should be collected into K2 EDTA tubes, kept on ice, and processed within 2 hours of collection to ensure peripheral blood mononuclear cells (PBMCs) do not lyse and release either DNA or RNA, which could interfere with subsequent analyses [17]. Published protocols for centrifugation of blood samples typically use two sequential spins (varying from 800 - 2000 g for 10-15 min) to separate plasma from buffy coat [18]. Our group first showed that a third bench top spin (1000 g for 5 min) is necessary to pellet any remaining cells, platelets and cellular debris [12]. Plasma and buffy coat containing PBMCs should be aliquoted and stored at -80 °C to avoid multiple freeze/thaw cycles. Although yields of cfDNA are reported to decline by approximately 30% per year in storage [19], we have successfully isolated cfDNA from plasma samples stored at -80°C for >12 years and analysed this by SNP 6.0 Array [14]. 

The findings here support previous studies [20], whereby either time delay or storage temperature of blood before centrifugation did not significantly affect levels of cfDNA. However, this variation in pre-analytical processing (centrifugation speed and time and temperature after blood drawing) can greatly affect miRNA profiles [21-23]. We suggest that the differences reported herein, which result from delayed processing post-venepuncture and higher centrifugation speeds, are largely due to altered recovery of platelets and microvesicles. Importantly, we have shown significant correlation between replicate samples, and with increasing centrifugation speed, but with significant reduction in the recovery of platelet-associated miRNAs (e.g. *hsa-miR-24, hsa-miR-191, hsa-miR-197* and *hsa-miR-223* [16]) after centrifugation at 10000g. Based on these findings, we recommended that blood samples are processed within 2 hours of venepuncture, with centrifugation speed carefully selected for the desired downstream analysis (i.e. platelet-rich vs. platelet-poor plasma). We concur with McDonald et al. [22] that future studies of circulating miRNA should explicitly state the centrifugation protocol and blood storage time to ensure reproducibility. 

A number of different manufacturer’s extraction kits are available for the isolation of circulating nucleic acids. We have shown that the chosen kit can have a profound effect on cfDNA yield, where efficient recovery is desired for down-stream analyses, including SNP 6.0 array [14] and deep-sequencing. Our results from spike-in experiments illustrate that the kits tested show variable recovery of both shorter and longer DNA fragments. Sequential fractionation has shown that low molecular weight cfDNA fractions often harbour genetic aberrations indicative of tumour-derived DNA [2,3]. The results presented here show that it is possible to efficiently recover cfDNA using commercially available kits, and targeted sequencing demonstrated efficient recovery of low molecular weight fractions, with detection of single nucleotide variants in cfDNA indicative of a tumour origin. There was little difference in recovery of cfDNA using the “top 2” kits as demonstrated by equivalent variant depth across 207 amplicons (average size 154 bp) using the Ion AmpliSeq™Cancer Hotspot Panel v2.

Our study has highlighted that the yield of miRNA can also vary widely between kits, as suggested previously [24]. Of note, although the *miRvana* isolation kit gave the lowest yields from 1 ml of plasma, ΔC_T_ values were significantly correlated with the results obtained using the miRNeasy Serum/Plasma kit and 200 µl of plasma. This kit has been used successfully to analyse miRNA profiles in a number of other studies [25-27]. We have also demonstrated good correlation between miRNA profiles of paired samples on two PCR independent array platforms. Together these results suggest that comparison of data generated from different extraction methods and analysis platforms is possible as long as the blood and plasma samples have been processed to the same protocol. In this study, we showed that RNA yield and recovery of the spike-in did not increase significantly with increasing plasma volume, suggesting that the columns become saturated above 200µl of plasma and are only capable of isolating a limited quantity of miRNA [28,29]. One recent study suggested that extraction from larger volumes of plasma increases the abundance of blood-borne inhibitors of qRT-PCR [30], which could also have contributed to the results obtained here. Extraction of miRNA from 200 µl of plasma is recommended to reduce the volume involved, minimise blood-borne inhibitors of qRT-PCR and enable replicate extractions from the same sample. 

The use of microRNA PCR array cards is common in the analysis of circulating miRNAs [31] and has generated promising results. We have previously analysed cfDNA from plasma samples stored for >12 years at -80 °C by whole genome approaches [14] but to our knowledge there are no prior reports of recovery of miRNA from samples stored for this length of time. We have shown here that retrospective analysis of circulating miRNAs by PCR array is possible, and that results correlated significantly with freshly prepared plasma. 

## Conclusion

In conclusion, we have shown that careful consideration of pre-analytical processing is required for optimum preparation of plasma for CNA analyses. The DNA Blood Mini kit and Circulating Nucleic Acids kit gave consistently higher yields and more efficient recovery of cfDNA than the other kits tested, with equivalent results detected by targeted next generation sequencing on the Ion PGM platform. The miRNeasy Serum/Plasma and *miRvana* kits gave consistent and reproducible results for miRNA isolation from as little as 200 µl plasma volume. It was also possible to analyse long term stored samples, which could enable comparison with other biomarker data obtained for samples in existing stored biobanks. However, in the era of the liquid biopsy, care must be taken in interpretation of miRNA data where the effects of sample processing may be as significant as disease state.

## Supporting Information

Figure S1 **Bioanalyser analysis of cell-free RNA from different volumes of plasma.** Representative Bioanalyser traces of cell-free RNA obtained from 1 ml, 500 µl or 200 µl of plasma using the miRNeasy Serum/Plasma kit. Starting volumes are highlighted above the trace and representative gels are shown below each trace. Quantities obtained are shown in the bar graph. Error bar = ±SEM, n = 3.
(TIF)

Figure S2 **Analysis of miRNA profiles on two different PCR array platforms and effect of long term storage on miRNA yield.** A. Two-tailed Spearman’s correlation analysis of miRNAs detected in 200 µl of plasma samples using the TLDA cards A & B (v2.0). miRNAs with C_T_ values >35 were not included in the analysis. B. Two-tailed Spearman’s correlation analysis of miRNAs detected in 200 µl of plasma samples using the Exiqon miRCURY LNA PCR platform. miRNAs with C_T_ values >35 were not included in the analysis. C. Two-tailed Spearman’s correlation analysis of ΔC_T_ values for miRNAs common to both TLDA and miRCURY LNA platforms. miRNAs with C_T_ values >35 were not included in the analysis. D. Representative miRCURY LNA PCR array amplification curves (right hand panels) and melt curves (left hand panels) of miRNAs with one melt temperature (Tm) (upper panels) and >1 Tm (lower panels). E. Two-tailed Spearman’s correlation analysis of C_T_ values for miRNAs detected in 200 µl of metastatic plasma samples comparing 10 pooled freshly extracted samples, with 10 stored for >12 years. The 95% confidence interval is shown on the graph (dotted lines). In total, 140 miRNAs were common to both samples. F. Spearman’s correlation analysis of ΔC_T_ values for miRNAs described in E.
(TIF)

Table S1 **Number of reads from two sequencing runs for 4 sets of paired cfDNA samples.** This table relates to Figure 2. QIA = QIAamp^®^ DNA Blood Mini kit; CNA = QIAamp^®^ CNA Kit.
(DOC)

Table S2 **Variants called for the different cfDNA samples.** This table relates to Figure 2. QIA = QIAamp^®^ DNA Blood Mini kit; CNA = QIAamp^®^ CNA Kit.
(DOC)

Table S3 **C_T_ and ΔC_T_ values obtained from plasma centrifuged at different speeds.** C_T_ and ΔC_T_ values obtained from TLDA A cards for miRNAs extracted from 200 µl of plasma, which had been centrifuged at either 1000g, 2000g or 10000g. This table relates to Figure 4C - E.
(XLS)

Table S4 **C_T_ and ΔC_T_ values obtained from fresh plasma and plasma stored for >12 years.** C_T_ and ΔC_T_ values obtained from TLDA A cards for miRNAs extracted from 200 µl of plasma from metastatic breast cancer patients, which had been freshly extracted or stored at -80 °C for >12 years. This table relates to Figure S2E & F.
(XLS)
