# Supplementary figures and images for: Influence of Plasma Processing on Recovery and Analysis of Circulating Nucleic Acids
Source: PLoS One. 2013 Oct 18;8(10):e77963. doi: 10.1371/journal.pone.0077963 (PMC3799744; doi:10.1371/journal.pone.0077963)

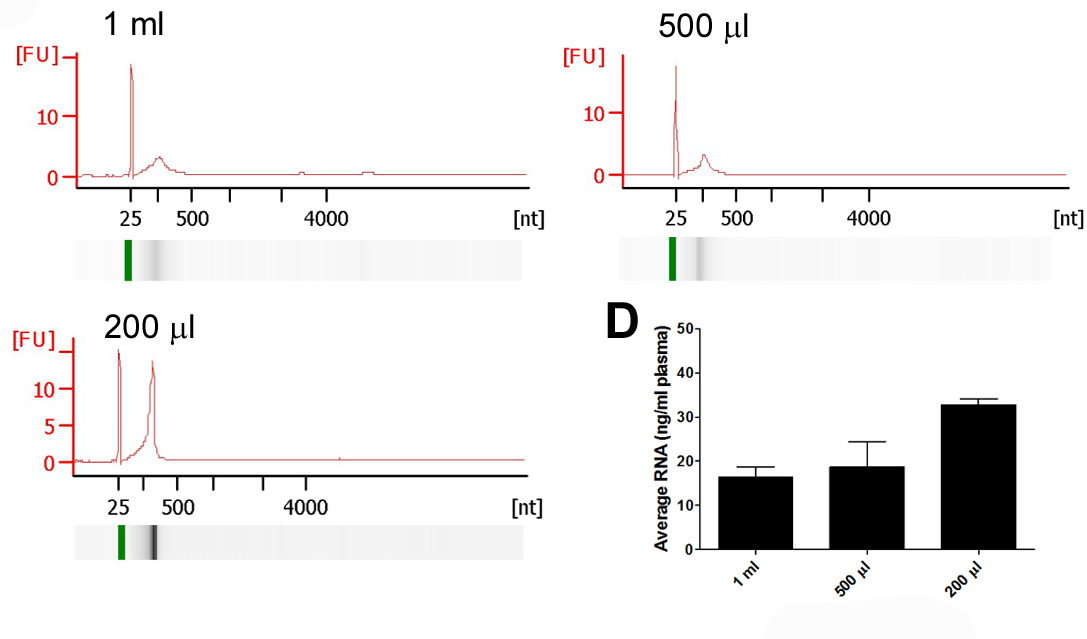

Supplement: Figure S1 — Bioanalyser analysis of cell-free RNA from different volumes of plasma. Representative Bioanalyser traces of cell-free RNA obtained from 1 ml, 500 µl or 200 µl of plasma using the miRNeasy Serum/Plasma kit. Starting volumes are highlighted above the trace and representative gels are shown below each trace. Quantities obtained are shown in the bar graph. Error bar = ±SEM, n = 3. (TIF) [file pone.0077963.s001.tif]

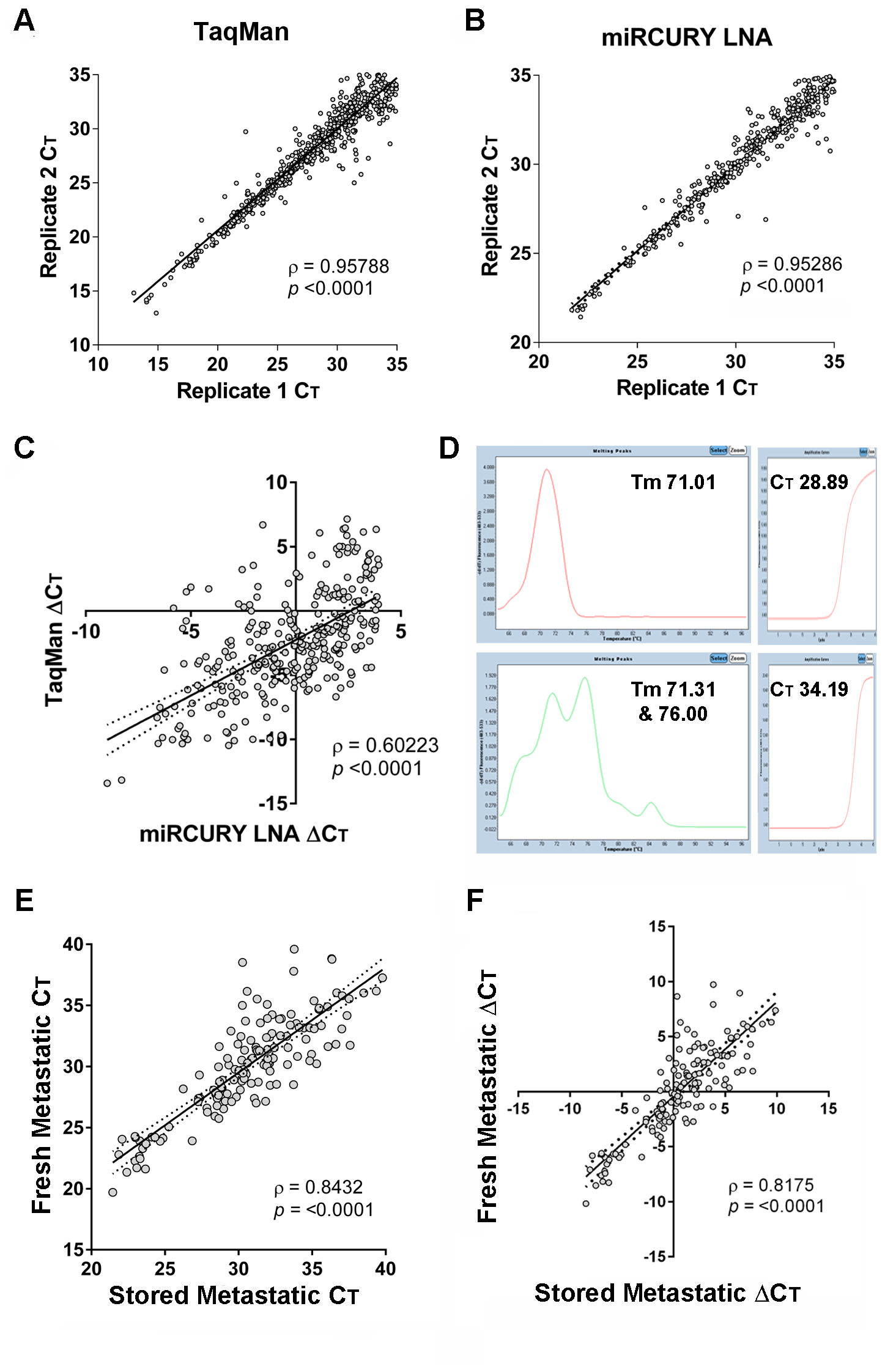

Supplement: Figure S2 — Analysis of miRNA profiles on two different PCR array platforms and effect of long term storage on miRNA yield. A. Two-tailed Spearman’s correlation analysis of miRNAs detected in 200 µl of plasma samples using the TLDA cards A & B (v2.0). miRNAs with CT values >35 were not included in the analysis. B. Two-tailed Spearman’s correlation analysis of miRNAs detected in 200 µl of plasma samples using the Exiqon miRCURY LNA PCR platform. miRNAs with CT values >35 were not included in the analysis. C. Two-tailed Spearman’s correlation analysis of ΔCT values for miRNAs common to both TLDA and miRCURY LNA platforms. miRNAs with CT values >35 were not included in the analysis. D. Representative miRCURY LNA PCR array amplification curves (right hand panels) and melt curves (left hand panels) of miRNAs with one melt temperature (Tm) (upper panels) and >1 Tm (lower panels). E. Two-tailed Spearman’s correlation analysis of CT values for miRNAs detected in 200 µl of metastatic plasma samples comparing 10 pooled freshly extracted samples, with 10 stored for >12 years. The 95% confidence interval is shown on the graph (dotted lines). In total, 140 miRNAs were common to both samples. F. Spearman’s correlation analysis of ΔCT values for miRNAs described in E. (TIF) [file pone.0077963.s002.tif]
