# Supplementary material for: Influence of Plasma Processing on Recovery and Analysis of Circulating Nucleic Acids
Source: PLoS One. 2013 Oct 18;8(10):e77963. doi: 10.1371/journal.pone.0077963 (PMC3799744; doi:10.1371/journal.pone.0077963)
Supplement: Table S1 — Number of reads from two sequencing runs for 4 sets of paired cfDNA samples. This table relates to Figure 2. QIA = QIAamp® DNA Blood Mini kit; CNA = QIAamp® CNA Kit. (DOC) [file pone.0077963.s003.doc]

**Table S1**

| **Sample** | **Total no. of bases (Mb)** | **Total no. of reads** | **Mapped Reads** | **Percentage mapped reads** | **Coverage depth (in fold)** | **Correlation value (ρ)** | **Slope** |
| --- | --- | --- | --- | --- | --- | --- | --- |
| P1 - QIA | 54 | 532,603 | 521,306 | 97.88% | 2,240 | 0.974 | 0.924 |
| P1 - CNA | 55 | 534,888 | 523,255 | 97.83% | 2,167 |
| P2 - QIA | 56 | 544,671 | 535,849 | 98.38% | 2,340 | 0.9585 | 0.667 |
| P2 - CNA | 55 | 527,451 | 517,337 | 98.08% | 2,202 |
| P3 - QIA | 48 | 483,443 | 472,872 | 97.81% | 1,816 | 0.860 | 0.507 |
| P3 - CNA | 51 | 490,864 | 479,056 | 97.59% | 1,974 |
| P4 - QIA | 41 | 401,101 | 390,798 | 97.43% | 1,490 | 0.963 | 1.088 |
| P4 - CNA | 52 | 515,393 | 498,894 | 96.80% | 1,884 |
| **Average** | **52** | **503,802** | **492,421** | **97.73%** | **2,014** | **---** | **---** |
| **Std dev** | **5** | **46,735** | **46,632** | **---** | **280** | **---** | **----** |
