# Supplementary material for: Influence of Plasma Processing on Recovery and Analysis of Circulating Nucleic Acids
Source: PLoS One. 2013 Oct 18;8(10):e77963. doi: 10.1371/journal.pone.0077963 (PMC3799744; doi:10.1371/journal.pone.0077963)
Supplement: Table S2 — Variants called for the different cfDNA samples. This table relates to Figure 2. QIA = QIAamp® DNA Blood Mini kit; CNA = QIAamp® CNA Kit. (DOC) [file pone.0077963.s004.doc]

**Table S2**

| **Sample ID** | **Total no. of variant called** | **No. COSMIC mutations called** | **No. of variants common to paired samples** | **Unique variants with frequency >10%** | **QIA vs CNA:**  **Wilcoxon signed ranks test p-value** |
| --- | --- | --- | --- | --- | --- |
| P1 - QIA | 20 | 4 | 17 | --- | **0.7820** |
| P1 - CNA | 18 | 3 |  | --- |  |
| P2 - QIA | 17 | 2 | 14 | --- | **0.426** |
| P2 - CNA | 15 | 2 |  | 1 |  |
| P3 - QIA | 37 | 6 | 19 | 2 | **0.002** |
| P3 - CNA | 21 | 2 |  | 2 |  |
| P4 - QIA | 20 | 3 | 18 | 1 | **0.5171** |
| P4 - CNA | 19 | 3 |  | 1 |  |
